# Characterization of *Cronartium ribicola* dsRNAs reveals novel members of the family *Totiviridae* and viral association with fungal virulence

**DOI:** 10.1186/s12985-019-1226-5

**Published:** 2019-10-17

**Authors:** Jun-Jun Liu, Yu Xiang, Richard A. Sniezko, Anna W. Schoettle, Holly Williams, Arezoo Zamany

**Affiliations:** 1 Pacific Forestry Centre, Canadian Forest Service, Natural Resources Canada, Victoria, BC V8Z 1M5 Canada; 2 Summerland Research and Development Centre, Agriculture and Agri-Food Canada, Summerland, BC V0H 1Z0 Canada; 3 Dorena Genetic Resource Center, USDA Forest Service, Cottage Grove, Oregon 97424 USA; 4 USDA Forest Service, Rocky Mountain Research Station, 240 West Prospect Road, Fort Collins, CO 80526 USA

**Keywords:** *Cronartium ribicola*, Double-stranded RNA, Five-needle pines, *Totiviridae*, White pine blister rust

## Abstract

**Background:**

Mycoviruses were recently discovered in the white pine blister rust (WPBR) fungus *Cronartium ribicola* (J.C. Fisch.). Detection and characterization of their double stranded RNA (dsRNA) would facilitate understanding of pathogen virulence and disease pathogenesis in WPBR systems.

**Methods:**

Full-length cDNAs were cloned from the dsRNAs purified from viral-infected *C. ribicola*, and their cDNA sequences were determined by DNA sequencing. Evolutionary relationships of the dsRNAs with related mycoviruses were determined by phylogenetic analysis. Dynamic distributions of the viral RNAs within samples of their fungal host *C. ribicola* were investigated by measurement of viral genome prevalence and viral gene expression.

**Results:**

In this study we identified and characterized five novel dsRNAs from *C. ribicola*, designated as *Cronartium ribicola* totivirus 1–5 (CrTV1 to CrTV5). These dsRNA sequences encode capsid protein and RNA-dependent RNA polymerase with significant homologies to dsRNA viruses of the family *Totiviridae*. Phylogenetic analysis showed that the CrTVs were grouped into two distinct clades. CrTV2 through CrTV5 clustered within the genus *Totivirus*. CrTV1 along with a few un-assigned dsRNAs constituted a distinct phyletic clade that is genetically distant from presently known genera in the *Totiviridae* family, indicating that CrTV1 represents a novel genus in the *Totiviridae* family. The CrTVs were prevalent in fungal samples obtained from infected western white pine, whitebark pine, and limber pines. Viral RNAs were generally expressed at higher levels during *in planta* mycelium growth than in aeciospores and urediniospores. CrTV4 was significantly associated with *C. ribicola* virulent pathotype and specific *C. ribicola* host tree species, suggesting dsRNAs as potential tools for dissection of pathogenic mechanisms of *C. ribicola* and diagnosis of *C. ribicola* pathotypes.

**Conclusion:**

Phylogenetic and expression analyses of viruses in the WPBR pathogen, *C. ribicola,* have enchanced our understanding of virus diversity in the family *Totiviridae*, and provided a potential strategy to utilize pathotype-associated mycoviruses to control fungal forest diseases.

## Background

The investigation of viruses that infect pathogenic fungi is important to understand interactions of plant-fungal pathogen-mycovirus. Infection of fungi by mycoviruses may result in no symptoms or in phenotypic alteration to the fungi, such as hypovirulence or hypervirulence. Elucidation of the mechanisms underlying virus-mediated hypovirulence or hypervirulence facilitates development of biological control tools for effective management of pathogenic fungi and parasites in plant and animal health [1–3].

The *Totiviridae* family is characterized by a double-stranded RNA (dsRNA) genome of 4–7 kilobases encapsidated in an icosahedral virus particle, about 40 nm in diameter. The virus genome often contains two overlapping open reading frames (ORF), encoding the viral capsid protein (CP) and RNA-dependent RNA polymerase (RdRp) [3]. To date, the virus family includes five approved genera, namely *Giardiavirus*, *Leishmaniavirus*, *Trichomonasvirus*, *Totivirus* and *Victorivirus* [4, 5]. Viruses in the genera *Totivirus* and *Victorivirus* mainly infect fungi while *Giardiavirus*, *Leishmaniavirus* and *Trichomonasvirus* are commonly present in protozoa. Two additional genera were recently proposed, including *Artivirus* with arthropod and fish hosts [6], and *Insevirus* with insect hosts [7]. Several totiviruses, mainly the members of genus *Totivirus*, have been reported in obligate biotrophs such as powdery mildew (ascomycetes) and rust fungi (basidiomycetes) [8, 9]. Further, some totiviruses (genus *Totivirus*) have also been detected in higher plants, including black currant (Ribes cultivars) [10].

*Cronartium ribicola* (J.C. Fisch) infects five-needle pine species of all ages, causing white pine blister rust (WPBR) disease in forests world-wide. The fungus was accidently introduced into western North America in 1910, killing 95% or more of white pine trees in the most seriously affected stands [11]. *C. ribicola* requires two host plant species to complete its life cycle, including development of pycniospore and aeciospore stages in five-needle pines, and development of urediniospore, teliospore, and basidiospore stages on an alternate host, principally Ribes. There are several virulent *C. ribicola* pathotypes detected in North America, and evidence suggests cytoplasmic inheritance of virulent traits [12], but molecular and genetic mechanisms underlying fungal pathogenicity and virulence are poorly understood in WPBR pathosystems. The factors that contribute to *C. ribicola* virulence and the severity of WPBR disease on susceptible five-needle pines are also not well understood [12, 13]. RNA viruses are found in various fungal pathogen species and are considered one of the important factors influencing disease development in plants following infection by a fungal pathogen [3]. Like other fungi, *C. ribicola* is susceptible to virus infection; we recently detected five novel mitoviruses in this species [14]. An investigation into mycovirus prevalence and phenotypic impact on virulence of *C. ribicola* may provide an effective approach to dissect white pine-blister rust interactions.

In the present work we report detection and characterization of novel viral dsRNAs in *C. ribicola*. Five new dsRNA viruses were discovered, and based on their genomic sequences and organization, four were designated as new members of the genus *Totivirus*. The fifth dsRNA virus belongs to a novel and previously unclassified genus of the family *Totiviridae*. The prevalence of the mycoviruses and their RNA expression were examined in various *C. ribicola* samples. One of the viruses was found to be significantly associated with *C. ribicola* virulence.

## Methods

### Collection of *C. ribicola* samples

Fungal samples were collected at aeciospore, urediniospore, or *in planta* mycelium growth stages. Aeciospores were collected from intact aecial blisters occurring on western white pine (*Pinus monticola*, Douglas ex D. Don) stems in the Vancouver Island (British Columbia-BC, Canada) and the Cottage Grove regions (Oregon-OR, USA). Black currant plants (*Ribes nigrum*, cultivar Ben Nevis) were inoculated using aeciospores of BC samples in a greenhouse at the Canadian Forest Service (CFS), Victoria, BC. Urediniospores (containing some mycelia) were collected from the undersides of infected Ribes leaves when the spores began to develop on currant plants. Infected tree tissues of *P. monticola* and limber pine (*P. flexilis*) inoculated at Dorena GRC, and whitebark pine (*P. albicaulis*) inoculated at CFS-Victoria were collected as fungal samples during the *in planta* growth stage of the rust lifecycle [13]. There was no restriction to sampling aeciospores in Canada and USA. A permit was issued by the Canadian Food Inspection Agency (CFIA) for CFS to import *C. ribicola* samples from OR, USA. Two fungal pathotypes (avirulent *avcr2* and virulent *vcr2*), isolated from *P. monticola* trees with the susceptible genotype (*cr2/cr2*) and the resistant genotype (*Cr2/Cr2* or *Cr2/cr2*), respectively; were included in the present study. A total of 33 *C. ribicola* samples (Additional file 5: Table S1), including six *vcr2* samples, were harvested, frozen in liquid nitrogen and immediately stored at − 80 °C before RNA extraction.

### dsRNA detection and purification

Total RNA was extracted from aeciospores and cankered stems of white pines as previously described [13], then treated with Turbo DNase I (Thermo Fisher Scientific), and nuclease S1 at 37 °C. The effect of enzymatic digestion was checked by agarose gel electrophoresis in TAE buffer (40 mM Tris base, 20 mM acetic acid, 1 mM EDTA, pH 8.0) and the gel was stained by ethidium bromide (0.5 μg/mL). Enzyme-treated dsRNA was purified again using an RNeasy Mini kit (Qiagen) before cDNA synthesis.

### cDNA cloning and sanger sequencing analysis

Totivirus-like contigs were discovered in the de novo assembly of a *C. ribicola* RNA-seq dataset [13] by Blastx searching against the Viral RefSeq database (viral.1.protein.faa.gz) downloaded from the National Center for Biotechnology Information (NCBI, Bethesda, Maryland, USA) (Additional file 5: Table S2). The virus-related contigs were used to design primers for cDNA cloning of the full virus genomes (Additional file 5: Table S3).

The dsRNA molecules purified from *C. ribicola* urediniospores and cankered western white pine stem samples (Additional file 5: Table S1) was used as templates for cDNA synthesis using a SuperScript III First-Strand Synthesis System (Life Technologies; Burlington, ON). The 5′- and 3′-ends of the viral genomes were determined by the method of rapid amplification of complementary DNA ends (RACE) using a 5′-RACE System (Invitrogen, Grand Island, NY). Following separation of RT-PCR products on a 1% (w/v) agarose gel, DNA fragments were purified from gel using a MinElute gel extraction kit (Qiagen) and cloned into the pGEM-T Easy vector (Promega). Recombinant plasmids were purified using a QIAprep Spin Miniprep kit (Qiagen) and the sequences of the inserts were determined by Sanger sequencing using T7 and SP6 universal primers, or internal primers as needed, on an ABI 3730XL sequencer.

DNA sequences were assembled into overlapping contigs for each individual viral genome using the software Sequencer (Gene Codes, Ann Arbor, MI). Complete nucleotide sequences of the viral assemblies were deposited in GenBank under accession numbers MK967418-MK967422. Open reading frames (ORFs) were determined using the NCBI ORF Finder. Protein domains and motifs were revealed by searching against the NCBI conserved domain database (CDD) [15] and the Pfam protein domain database at EMBL-EBI [16]. Multiple sequence alignment of protein sequences was conducted and identities between the sequences were calculated using Clustal-Omega at EMBL-EBI website. On the basis of the aligned sequences, phylogenetic trees were constructed using the statistical method of maximum likelihood (ML) with the MEGA version 6.0 program [17]. A pseudoknot structure in the viral genomes was predicted using the HPknotter program [18].

### Prevalence of viral genomes across fungal samples assessed by quantitative reverse transcription PCR (qRT-PCR)

Virus prevalence was evaluated in 33 rust samples, including three host plants infected by *C. ribicola*. Two micrograms of total RNA, including viral dsRNA genomes and ssRNA transcripts or replication intermediates, were used to synthesise the first strand cDNA with random primers using a Superscript VILO master mix kit (Life Technologies). Viral genome-specific primers were designed at 3′-terminal regions showing no homology to the other four viral dsRNA genomes using the software PrimerExp (ABI) (Additional file 5: Table S3), and qRT-PCR was run with three replicates per sample on an Applied Biosystems 7500 Fast Real-time PCR System (Life Technologies). Melt curve analysis showed a single sharp peak for each reaction, indicating target-specific amplification of a unique amplicon for each virus. No cross-reactions against the rest of the viruses were observed for each pair of PCR primers. *C. ribicola* and *P. monticola* genomic DNA, total RNA with no reverse transcriptase, and water were included as negative controls for each viral genome on each plate. The *C. ribicola α-tubulin* gene was included as an internal control for normalization of fungal RNA levels across the fungal samples.

The 2^−ΔΔCT^ method was used to calculate relative RNA level using Expression Suite Software v1.0.3 (Life Technologies) [19]. RNA levels were compared between fungal samples, and significance of the differences were determined using one-tailed independent t-tests (*P* < 0.05).

### Assessment of viral RNA levels by RNA-seq analysis

Relative levels of viral transcript-related RNAs were compared across different viral genomes as well as across different stages of the fungal life-cycle using RNA-seq data from previous transcriptome analyses [13, 20]. The RNA-seq cDNA libraries were constructed from total RNA with depletion of rRNAs using a TruSeq RNA-seq Sample Preparation Kit (Illumina, San Diego, CA, US). Transcriptomic data from RNA-seq analysis were thus presumably generated mainly from transcripts or ssRNA. Nine *avcr2* samples were available for viral data analysis (Additional file 5: Table S1), including three aeciospore samples (SRR1583540, SRR1583545, and SRR1583552), three urediniospore samples (SRR1583557-SRR1583559) and three western white pine cankered stem samples (SRR3273235-SRR3273237). In addition, three healthy western white pine stem samples (SRR1574690–1574692) were included as negative controls, and targeted viral transcripts were not detected in the RNA-seq data of these samples which were not infected by *C. ribicola*. CLC Genomics Workbench v5.5 was used for read mapping with parameter setting at a mismatch cost of 1, indel cost of 3, length fraction of 0.95, and similarity fraction of 0.95. Trimmed reads were mapped to viral genomes and only paired reads (fragments) were counted in mapping. Fragments per kilobase of exon per million reads mapped (FPKM) were used to evaluate the relative level of ssRNA(+) or transcripts. FPKM values were compared using one-tailed independent t-tests (*P* < 0.05) to assess differences in viral transcript levels among fungal samples or among different viruses inside the same fungal isolate.

## Results

### Detection of dsRNAs in *C. ribicola*

**Fig. 1:**
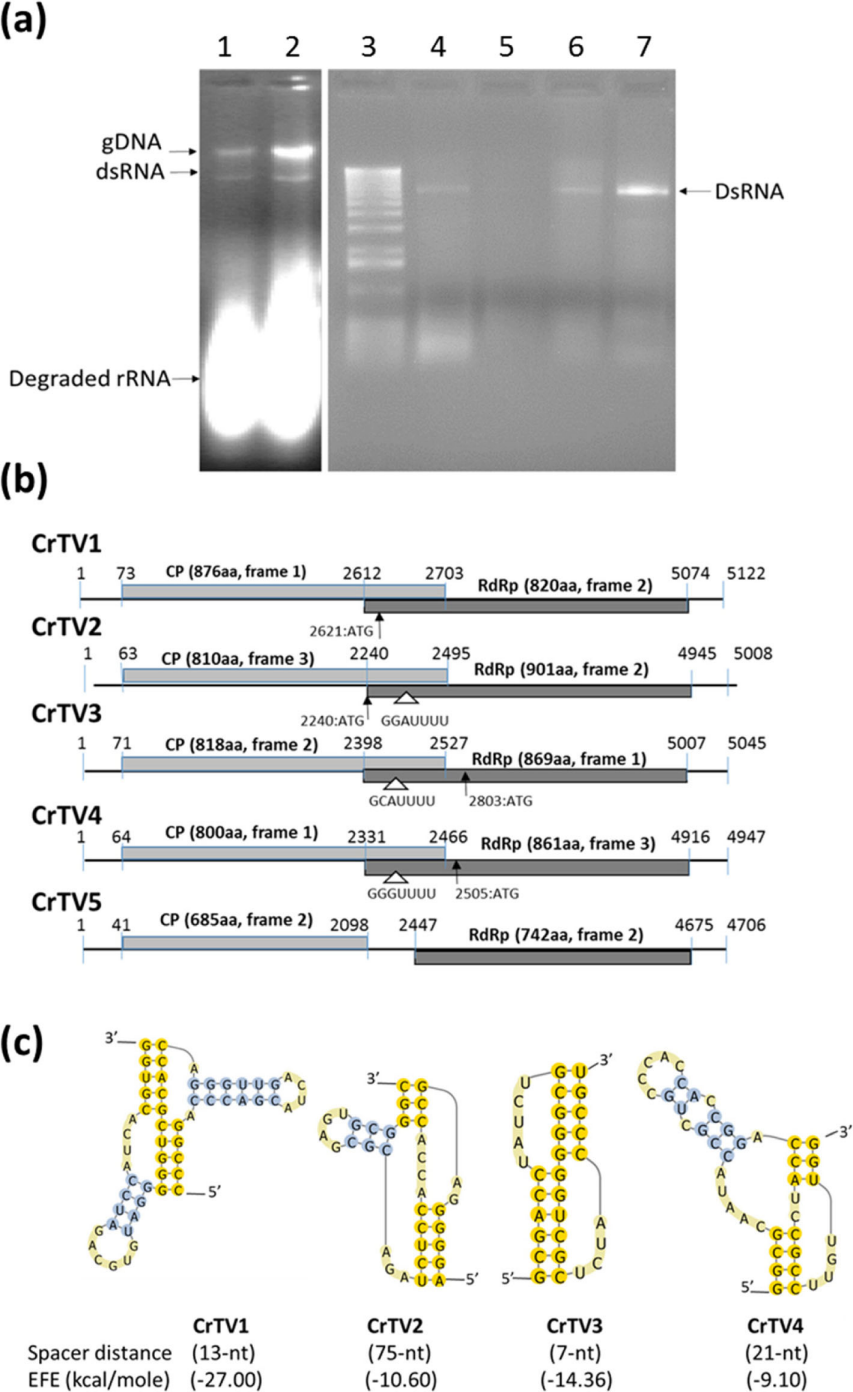
Analysis of the full-length genomes of CrTVs. **a** Agarose gel electrophoresis of dsRNA extracted from *Cronartium ribicola* spores and cankered stems of western white pine. Two cankered western white pine stem samples (lane 1–2) shows presence of dsRNA with genomic DNA and partially degraded rRNAs without enzyme treatment. Three of four spore samples (lane 3: DNA standard ladder; lane 4–5: urediniospores; lane 6–7: aeciospores) show obvious presence of dsRNA after treatment with DNase I, nuclease S1. **b** Diagrammatic representation of CrTV genome organization. Open reading frame (ORF) positions, reading phases, and putative protein lengths are labelled. ORF1 and ORF2 encode putative capsid protein and RdRp respectively; and they overlap in four genomes CrTV1, CrTV2, CrTV3 and CrTV4. ORF2 is presumptively translated as a fusion protein with ORF1 through a + 1 ribosomal frameshift for CrTV1, or through a − 1 ribosomal frameshift for CrTV2, CrTV3, and CrTV4. **c** Putative pseudoknots were predicted using the HPknotter program and viewed using the PseudoViewer3 program. Spacer distances were calculated from the pseudoknot upstream to the potential slippery site for CrTV2, CrTV3, and CrTV4, or upstream to the in-frame stop codon of ORF2 for CrTV1. Minimal free energy (MEF) are presented

Double-strand RNA (dsRNA) was detected in *C. ribicola* aeciospores, urediniospores, and *P. monticola* stems infected by *C. ribicola* with cankered disease symptoms (Additional file 5: Table S1). After treatment of total RNAs with DNase and nuclease S1, clear dsRNA fragments were estimated with a size of about 5 Kb by agarose gel electrophoresis (Fig. 1a). Purified dsRNA samples were then used for cDNA cloning and Sanger DNA sequencing.

### Full-length genome sequences of the dsRNA viruses infecting *C. ribicola*

Our previously assembled *C. ribicola* transcriptome [13] was used to search for candidate dsRNA sequences by a Blast analysis. A Blastx search revealed several *C. ribicola* contigs with significant homology hits to the viral database. The top-hit viral species included Black raspberry virus F (BRV-F, 6.23E-05~ 3.36E-39), Saccharomyces virus L-A (SCV-LA, 5.08E-53), Xanthophyllomyces dendrorhous virus L1A (XDV-L1A, 8.21E-23~ 2.31E-120), and Persimmon latent virus (PeLV, 1.99E-12) (Additional file 5: Table S2). Because BRV-F, SCV-LA, and XDV-L1A are totiviruses and the *C. ribicola* dsRNA was in the size range of totiviru*s* genomes, these *C. ribicola* contig sequences were used to design oligonucleotide primers for cDNA cloning of the viral genomes (Additional file 5: Table S3).

Following RT-PCR, RACE, and cDNA cloning of the dsRNA (Fig. 1a), five independent sequences were determined by Sanger DNA sequencing. The full-length dsRNAs, from large to small, were 5112, 5045, 5008, 4947 and 4706 bp in length (Additional file 5: Table S4). All sequences contain two open reading frames (ORF1 and ORF2) as displayed in Fig. 1b and Additional file 5: Table S4; their full-length cDNA sequences are shown in Additional file 5: Table S5.

A domain search against the NCBI conserved domain database (CDD) found that ORF1 of dsRNA 2, 4, and 5 encoded putative proteins with significant homologies (E-values ranging from 3.44E-03 to 3.32E-87) to the major coat protein of SCV-LA, a typical totivirus, while ORF1 of dsRNA1 encoded a putative protein with limited homology to Marek’s disease glycoprotein A (E-value at 2.85E-03) (Additional file 5: Table S4). The ORF2 of all five dsRNAs had significant homologies to the reverse transcriptase (RT, RNA-dependent DNA polymerase)-like family (1.81E-10 ~ 8.75E-60) (Additional file 5: Table S4). A domain search against the Pfam at EMBL-EBI database revealed the ORF2 of all five dsRNAs encoded putative proteins with significant homologies (E-values ranging from 3.8E-14 to 5.4E-56) to viral RNA-directed RNA-polymerases of the RdRP_4 family (Additional file 5: Table S5). Blastx search against NBCI nr database indicated that the five dsRNAs respectively had the highest identities to Pythium polare RNA virus 2 (PpRV2, LC376045), Puccinia striiformis totivirus 2 (PsV2, KY207362), Phakopsora pachyrhizi mycovirus (PaMV, KT222801), PsV3 (KY207363), and PsV5 (KY207365) (Additional file 5: Table S6 and S7). Our phylogenetic and sequence homology data indicated that the five dsRNAs originated from totiviruses that infected the fungus *C. ribicola.* Therefore, we tentatively named the new fungal viruses *Cronartium ribicola* totivirus 1 to 5 (CrTV1 to CrTV5) (Fig. 1b).

### Genome organization of CrTVs

ORF1 and ORF2 overlapped in the genomes of CrTV1 to CrTV4, but were separated by a 349-bp internal sequence in the CrTV5 genome. CrTV2, CrTV3, and CrTV4 were predicted to be translated with ORF1 and ORF2 as a fusion protein through a − 1 ribosomal frameshift, supported by the presence of a GGGUUUU-like slippery site in the overlapping regions of ORF1 and ORF2 (Fig. 1b). This slippery site resembles the consensus XXXYYYZ motif presumed in the red-yeast totiviruses (XdV-LA1 and XdV-L2) and other viruses of genera *Totivirus* and *Giardiavirus* [21, 22]. A pseudoknot structure was predicted to form downstream of the putative slippery sites in the transcripts of CrTV2, CrTV3 and CrTV4 (Fig. 1c). The pseudoknot structure was proposed to pause the ribosomes, thus favoring frameshifting-translation of a fusion protein [23]. CrTV1 was predicted to be translated as a fusion protein with a rare + 1 ribosomal frameshift, like Leishmania RNA virus 1 (LRV1) of the genus *Leishmaniavirus*, in the *Totiviridae* family [24]. A putative pseudoknot structure was predicted in CrTV1, but no consensus slippery site was characterized for a + 1 ribosomal frameshift (Fig. 1c).

### Alignments of putative protein sequences and phylogeny of CrTVs

Both assigned and unassigned representative members of the family *Totiviridae* were included in an alignment analysis of protein sequences using Clustal Omega. CrTV1 to CrTV5 showed the highest identities to PpRV2 (CP at 22% and RdRp at 32%), PaMV (CP at 41% and RdRP at 52%), PsV2 (CP at 35% and RdRp at 47%), PsV3 (CP at 46% and RdRp at 56%), and PsV5 (CP at 46% and RdRP at 46%), respectively (Additional file 5: Table S6 and S7). Because our samples included urediniospores collected from Ribes, CrTVs were compared to an available RdRp fragment (AJ133821) of an unidentified Totiviridae genomic RNA from black currant [10], and we found that CrTV5 had the highest identity at 41.03% while CrTV1 showed the lowest identity at 13.91%. Protein sequence identities among the five CrTVs themselves were much lower, ranging from 8 to 34% for CPs, and from 15 to 47% for RdRps (Additional file 5: Table S6 and S7). This suggests that the CrTVs probably evolved from different ancestors.

**Fig. 2:**
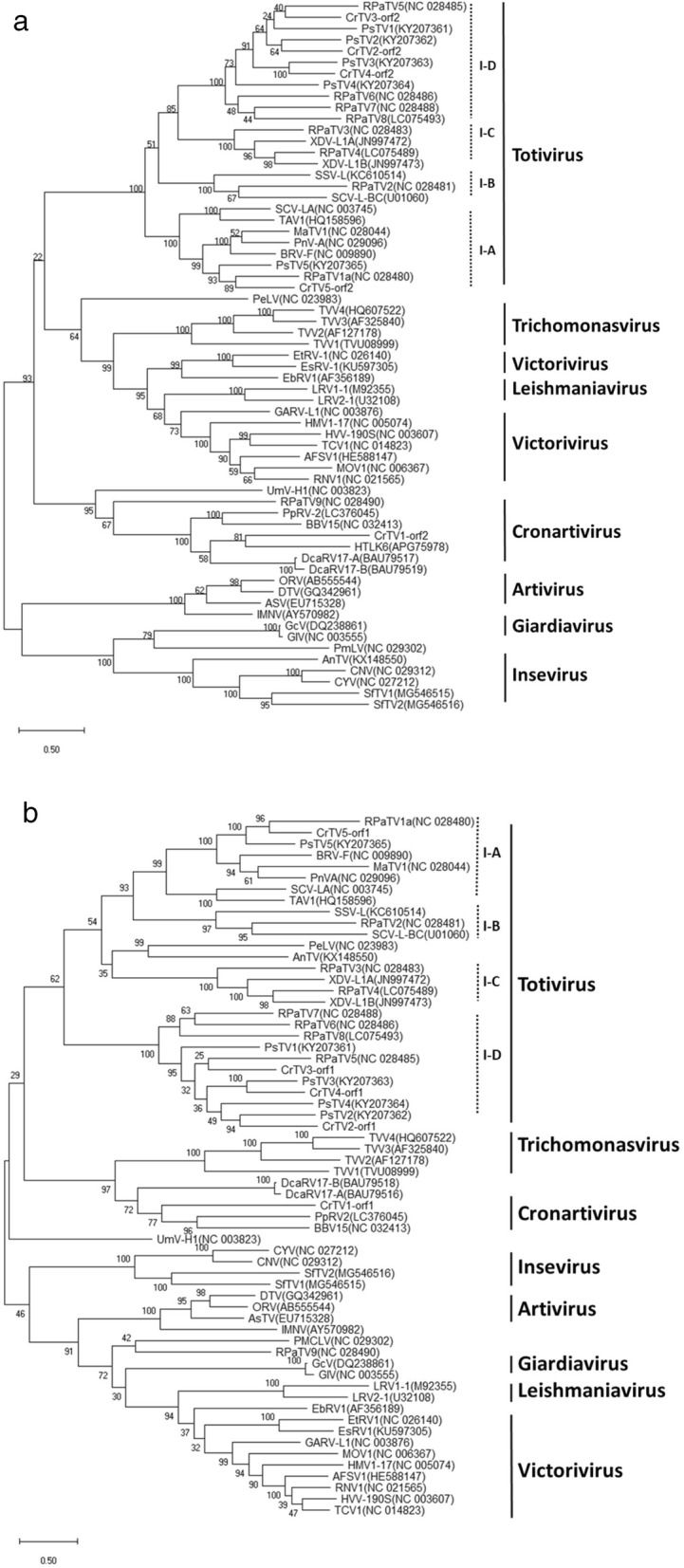
Phylogenetic trees for CrTVs and other members in the family *Totiviridae* based on the deduced amino acid sequences for the RdRp (**a**) and CP (**b**). Sequences were aligned using Clustal Omega and trees were generated using the maximum likelihood method with a bootstrap test of 100 iterations in MEGA 6 software. Five approved genera are labelled for the well-supported clades on each tree. Bootstrap support resulting from 100 replicates is shown on the internodes and branch lengths correspond to genetic distance; the scale bar at lower left corresponds to a genetic distance of 0.5 for the protein sequences. Virus sequences were downloaded from GenBank and presented here as: Anopheles totivirus (AnTV, KX148550), Armigeres subalbatus virus (AsTV, EU715328), Aspergillus foetidus slow virus 1 (AFSV-1, HE588147), Beihai barnacle virus 15 (BBV15, NC_032413), Black raspberry virus F (BRV-F, NC_009890), *Camponotus nipponicus* virus (CNV, NC_029312), *Camponotus yamaokai* virus (CYV, NC_027212), Diatom colony associated dsRNA virus 17 (DcaRV-17A, BAU79516; DcaRV-17B, BAU79518), *Drosophila melanogaster* totivirus (DTV, GQ342961), Eimeria brunetti RNA virus 1 (EbRV1, AF356189), Eimeria stiedai RNA virus 1 (EsRV-1, KU597305), Eimeria tenella RNA virus 1 (EtRV1, NC_026140), Giardia canis virus (GcV, DQ238861), *Giardia lamblia* virus (GlV, NC_003555), Gremmeniella abietina RNA virus L1 (GARV-L1, NC_003876), Helicobasidium mompa totivirus 1–17(HMV1–17, NC_005074), Helminthosporium victoriae virus 190S (HVV-190S, NC_003607), Hubei toti-like virus 6 (HTLV6, KX882940), Leishmania RNA virus 1 (LRV1–1, M92355), Leishmania RNA virus 2 (LRV2–1, U32108), Magnaporthe oryzae virus 1 (MOV-1, NC_006367), Maize-associated totivirus 1 (MaTV-1, NC_028044), Omono iiver virus (ORV, AB555544), Panax notoginseng virus A (PnV-A, NC_029096), Penaeid shrimp infectious myonecrosis virus (IMNV, AY570982), Persimmon latent virus (PeLV, NC_023983); Phakopsora pachyrhizi mycovirus (PaMV, KT222801), Piscine myocarditis-like virus (PMCLV, NC_029302), Puccinia striiformis totivirus (PsV1, KY207361; PsV2, KY207362; PsV3, KY207363; PsV5, KY207365), Pythium polare RNA virus 2 (PpRV2, LC376045), Red clover powdery mildew-associated totivirus (RPaTV1a, NC_028480;RPaTV2, NC_028481; RPaTV3, NC_028483; RPaTV4, LC075489; RPaTV5, NC_028485; RPaTV6, NC_028486; RPaTV7, NC_028488; RPaTV8, LC075493; RPaTV9, NC_028490), Rosellinia necatrix victorivirus 1 (RNV-1, NC_021565), *Saccharomyces cerevisiae* virus (SCV-L-BC, U01060; SCV-LA, NC_003745), Scheffersomyces segobiensis virus L (SSV-L, KC610514), *Sogatella furcifera* virus (SfTV1, MG546515; SfTV2, MG546516), Tolypocladium cylindrosporum virus 1 (TCV1, NC_014823), Trichomonas vaginalis virus (TVV1, TVU08999;TVV2, AF127178;TVV3, AF325840;TVV4, HQ607522), Tuber aestivum virus 1 (TAV1, HQ158596), *Ustilago maydis* virus H1 (UmV-H1, NC_003823), and Xanthophyllomyces dendrorhous virus (XDV-L1A, JN997472; XDV-L1B, JN997473)

Based on the alignments of the putative CP and RdRp sequences, phylogenetic trees were constructed using the ML method in MEGA (Fig. 2). Five approved genera of the *Totiviridae* family displayed well-supported clades in both CP- and RdRp-based phylogenetic trees (Fig. 2). The viruses in the genus *Totivirus* were grouped into four subclades (I-A to I-D), as previously denoted [8, 9]. CrTV2, 3, and 4 were clustered in pairs with PsV2, red clover powdery mildew-associated totivirus 5 (RPaTV5), and PsV3, respectively, inside the subclade I-D of the genus *Totivirus*, while CrTV5 was clustered with RPaTV1a and PsV5 inside the *Totivirus* subclade I-A. The phylogenetic relationships indicated that CrTV2 to CrTV5 are likely new species of the *Totivirus* genus.

In contrast, CrTV1 was distant from the other four CrTVs and did not associate with any previously reported genera. In the RdRp-based phylogenetic tree, CrTV1 clustered together with several other unclassified members, including Hubei toti-like virus 6 (HTLV6, KX882940), PpRV2 (LC376045), Beihai barnacle virus 15 (BBV15, NC_032413), Diatom colony associated dsRNA virus (DcaRV-17A and B, BAU79517 and BAU79519), RPaTV9 (NC_028490), *Ustilago maydis* virus H1 (UmV-H1, NC_003823), forming a separated phylogenetic branch clade. This clade was parallel to the other three major branches displayed in the RdRp-based phylogenetic tree (Fig. 2a), including one branch that contained only the genus *Totivirus*, another branch that was formed by the genera *Tricomonasvirus*, *Leishmaniavirus*, and *Victorivirus* (including eimeriaviruses as a potential subgenus of the genus) [25], and a third branch that was composed of the genera *Artivirus*, *Giardivirus* and *Insevirus* (Fig. 2a).

In the CP phylogenetic tree (Fig. 2b), CrTV1 clustered together with PpRV2 (LC376045) and BBLV15 (NC_032413) in a separate clade. The CP phylogenetic tree displayed four major branches that were parallel with each other. The CrTV1-containing clade was closely grouped with the clade of *Trichomonasvirus* in one major branch. The other three major branches included a clade of *Artivirus, Giardiavirus*, *Leishmaniavirus* and *Victorivirus*, a clade with the genus *Insevirus* only, and a clade with the genus *Totivirus* only. In general, CP genes between two groups of toti- and toti-like viruses are not well conserved. Thus, CP-based phylogenetic trees were separately created using two datasets, one for the members of genus Totivirus (except for UmV-HI) (Additional file 1: Figure S1-a) and DcaRV17/PpRV2 and UmV-HI related viruses, respectively (Additional file 1: Figure S1-b). Similar phylogenetic relationships were observed when the two groups were separated. Phylogenetic distances of CrTV1 and several other unclassified members to the characterized genera suggests the existence of another genus in the *Totiviridae* family which we tentatively named as *Cronartivirus* in this report.

### Prevalence of CrTVs affected by *C. ribicola* life cycle, genotypes, and host plants

**Fig. 3:**
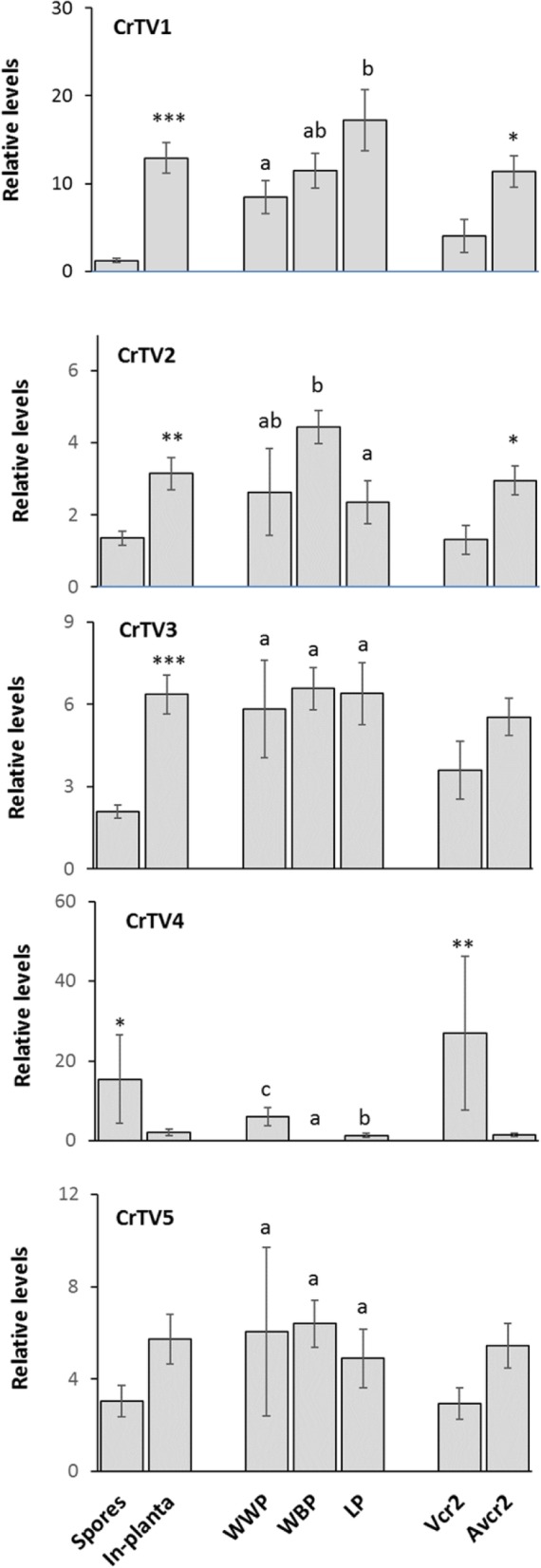
Viral RNA abundance of *Cronartium ribicola* totiviruses (CrTV1 to CrTV5) as measured by qRT-PCR analysis. Total RNA levels were normalized across 33 tested fungal samples using *C. ribicola* α tubulin transcript as the calibrator. Relative RNA levels were calculated using the 2^−ΔΔCt^ algorithm. Means for relative RNA levels of each virus genome were analyzed in three comparisons: spores vs. *in planta* mycelium growth in infected pine tissues; *in planta* mycelium growth in three host plants: WWP-western white pine (*Pinus monticola*), WBP-whitebark pine (*P. albicaulis*), and LP-limber pine (*P. flexilis*); and *C. ribicola* pathotypes *vcr2* vs. *avcr2*. Bars show average RNA levels of each virus with standard error of the mean (SEM). Different letters on the tops of the bars indicate significant difference (t-test, *P* < 0.05) among three host plants. One, two, and three stars (*) indicate *P* < 0.05, *P* < 0.01, and *P* < 0.001, respectively, in paired comparisons

The relative abundance of CrTV genomes was evaluated by qRT-PCR across 33 *C. ribicola* samples (Fig. 3). PCR primers were designed at the highly variable regions to distinguish different CrTV genomes (Additional file 5: Table S3). We used the *C. ribicola α-tubulin* gene as an internal control for normalization of total RNA extracted from different *C. ribicola* samples. No PCR product was detected in negative controls (including *C. ribicola* and *P. monticola* genomic DNA, total RNAs without reverse-transcriptase, and water). RNA-seq analysis also revealed the presence of CrTVs in cankered western white pines and absence in healthy pine stems. All of the available evidence indicated no integration of CrTVs into the genomes of *C. ribicola* and five-needle pines. qRT-PCR detected these totiviruses repeatedly in spore samples with dsRNA levels undetectable by agarose gel electrophoresis (Fig. 1a). Significant changes in genome abundance of CrTV1 through 4 during the life-cycle of *C. ribicola* were discovered. CrTV1, CrTV2, and CrTV3 showed higher levels during *in-planta* mycelium growth than during the aeciospore and urediniospore stages. CrTV4 showed an opposite pattern, with 7.5 times more abundance in fungal spores than at the *in planta* mycelium growth stage (15.423 ± 11.157 vs. 2.069 ± 3.661, t-test *P* = 0.03).

When viral abundance was compared between *C. ribicola* avirulent samples (*avcr2*) and virulent samples (*vcr2*), CrTV3 and CrTV5 showed no significant difference. However, CrTV1 and CrTV2 accumulated significantly more in *avcr2* than in *vcr2* (t-test, *P* < 0.05). In contrast, CrTV4 was on average 20 times more abundant in *vcr2* than in a*vcr2 samples* (t-test, *P* < 0.01) (Fig. 3). Pearson correlation analysis further demonstrated that abundance of CrTV4 was positively correlated to the virulence *of vcr2* samples(*R* = 0.5127, *P* = 0.002, *N* = 33 (Additional file 2: Figure S2).

We further examined the effects of *C. ribicola* host plants on the prevalence of CrTVs. No significant change was detected for the relative amounts of CrTV3 and CrTV5 genomes across the three host plants (*P. monticola*, *P. albicaulis*, and *P. flexilis*) infected by *C. ribicola*. In contrast, CrTV1 showed higher genome abundance in *P. flexilis* than in *P. monticola* while CrTV2 displayed higher genome abundance in *P. albicaulis* than in *P. flexilis* (Fig. 3). Of all five CrTVs, only CrTV4 showed a host range restriction. CrTV1, 2, 3 and 5 were detected in all 33 *C. ribicola* samples, but the CrTV4 genome was not detectable in all nine *C. ribicola-*infected *P. albicaulis* samples and three of nine *C. ribicola*-infected *P. flexilis* samples. CrTV4 was detected in all six cankered *P. monticola* samples with significantly higher levels than in infected *P. flexilis* (5.997 ± 2.276 vs. 1.290 ± 0.535, t-test, *P* = 0.014). CrTV4 accumulation across all 33 *C. ribicola* samples are shown in Additional file 3: Figure S3. In particular, *vcr2* aeciospores had a far higher CrTV4 abundance than all other sample types (one-way ANOVA, *P* = 0.014). Our qRT-PCR analysis results suggest that *C. ribicola* genotypes, developmental stages of the fungal life-cycle, and plant hosts are important factors affecting the prevalence of CrTVs.

### Differential expression of CrTV RNAs assessed by RNA-seq

**Fig. 4:**
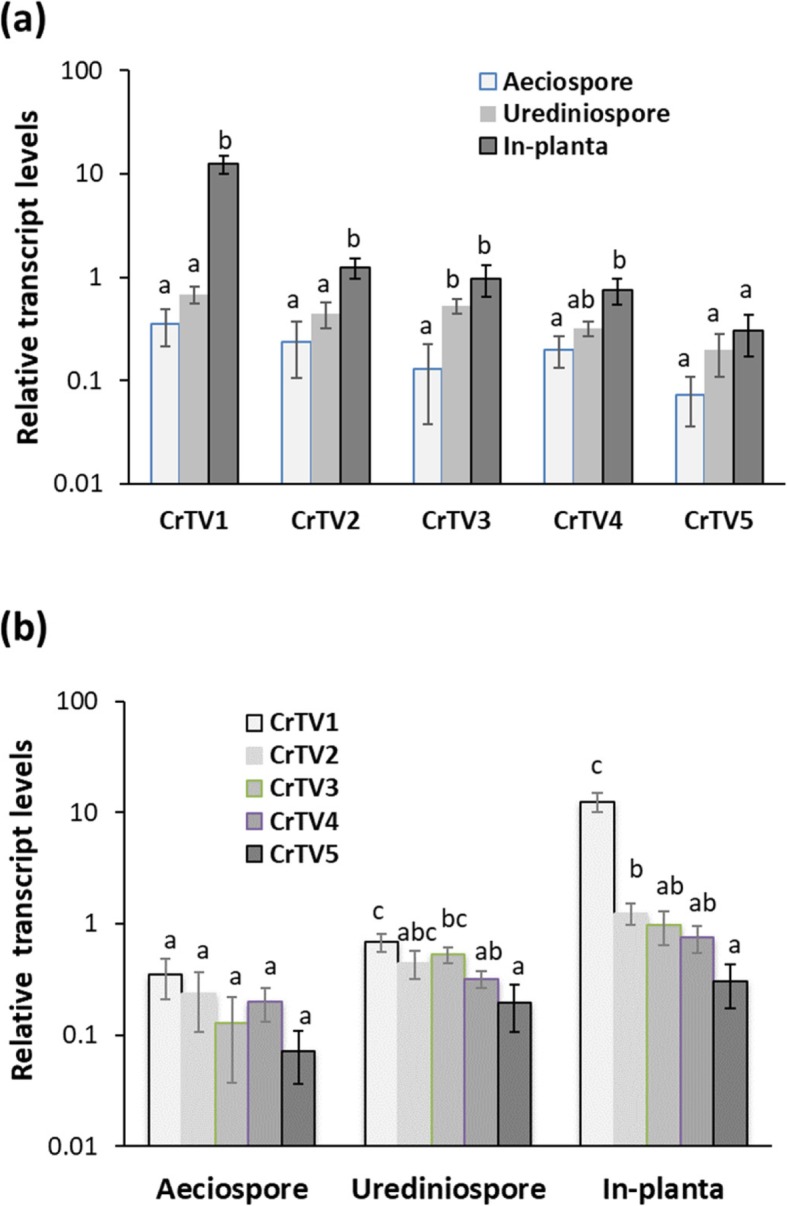
Relative levels of transcript-related RNAs of *Cronartium ribicola* totiviruses (CrTV1 to CrTV5) as measured by FPKM using RNA-seq data. **a** Relative RNA levels of each virus compared with three stages of the fungal life cycle (aeciospore, urediniospore, and *in planta* mycelium growth); **b** Relative RNA levels in each sample type compared with the five totiviruses in each of three sample types. Bars show average RNA levels of each virus with standard error of the mean (SEM). Different letters on the tops of the bars indicate significant differences (t-test, *P* < 0.05) between the samples or the viruses from the same fungal sample using t-test

RNA-seq-based FPKM was used to evaluate relative viral transcript-related RNA levels across three stages of the *C. ribicola* lifecycle as well as across five CrTVs (Fig. 4). All of the CrTVs except CrTV5 showed relative transcript-related RNA levels significantly higher at the *in planta* mycelium growth stage (fold changes ranging from 4 to 36) than that at the aeciospore stage (t-test, *P* < 0.05). Of the five CrTVs, only CrTV3 showed a significantly higher level of RNA expression in urediniospores than in aeciospores (Fig. 4b), suggesting a possible interaction between Ribes and *C. ribicola* that may affect the transcription and replication activity of CrTV3.

Because data were normalized across the transcriptome, FPKM can be used to compare transcript-related RNA levels among the five viruses in the same sample (Fig. 4b). At the aeciospore stage, all five CrTVs showed similar transcript-related RNA levels. In urediniospores, CrTV1 had the highest average transcript-related RNA level, which was significantly higher than that in CrTV4 and CrTV5, while CrTV2 and CrTV3 had transcript-related RNA at intermediate levels. At the *in planta* growth stage in cankered *P. monticola* stems with both *Cr2/−* and *cr2/cr2* genotypes, CrTV1 showed an average transcript-related RNA level 10 to 40 times higher than the other four viruses (t-test, *P* < 0.01), followed by CrTV2 with 4 times higher levels than CrTV5 (t-test, *P* < 0.05).

The accumulation profiles of CrTVs showed a significant correlation between the qRT-PCR and RNA-seq datasets for all five CrTVs across the nine *avcr2* samples assessed with both methods (Pearson Correlation Coefficient *R* = 0.47, *P* = 0.001, Additional file 4: Figure S4). CrTV1 showed a Pearson correlation at the highest level (*R* = 0.845, *P* = 0.004) and CrTV4 showed a correlation at an intermediate level (*R* = 0.631, *P* = 0.068) probably due to the lower abundance of CrTV4 RNA relative to CrTV1 and the limited sample size (*N* = 9).

## Discussion

The five CrTV dsRNA sequences discovered in *C. ribicola* shared similar genome sizes ranging from 4.7 to 5.1 kb, but the sequence homologies between them were limited. All five CrTV genomes contained two ORFs encoding unique CP and RdRp, putatively assigned to the family *Totiviridea*. We identified three types of genome organizations in the CrTVs with respect to ORF translation. CrTV2, CrTV3, and CrTV4 were predicted to have two overlapping ORFs; the RdRp is likely translated as a fusion protein with the CP as a consequence of a ribosomal − 1 frameshift event. A putative canonical slippery site with GGGUUUU-like sequences was further predicted in CrTV2, CrTV3, and CrTV4, which resembles the slippery site presumed in the red-yeast totivirus XdV-LA1 and XdV-L2 [21]. In contrast, CrTV1 RdRp was predicted to be translated as a fusion protein by a + 1 ribosomal frameshift, similar to Leishmania RNA virus 1 and a number of other members of the family *Totiviridae* [26–30]. A canonical slippery site is required for a + 1 ribosomal frameshift, but none has been confirmed in the family *Totiviridae* yet. CrTV5 represented a different type of genome organization, with its CP and RdRp separated by an internal non-translated sequence. This pattern was also observed in the Helminthosporium victorivirus [31], and the myonecrosis virus (IMNV, AY570982) that infects penaeid shrimp [32].

With advancements in next generation sequencing (NGS) technology and wide application of metagenomics, totiviruses have been sequenced from fungi, protozoa, arthropods, insects, plants, and fish. Small RNAs and ribosomal RNA depleted total RNA are commonly used for NGS-based viral RNA discovery [33]. NGS does not require any prior knowledge for identification of novel viruses in field samples [34], especially those previously overlooked by traditional virus diagnosis tools [35]. More recently, transcriptome sequencing was used for detection of viral agents in fungi and higher plants, such as citrus [36]. Our previous work detected several mitoviruses in *C. ribicola* using RNA-seq-based NGS [14]. In the present study, the same transcriptomic assemblies were used to search the NCBI Blast database, facilitating discovery of the CrTVs. These studies showed that sequencing of three transcriptomes in one sample allows simultaneous identification of novel RNA sequences originating from plant, fungi, and mycoviruses in fungal infected plant tissues. Co-existence of RNA viruses with different genome organizations may prevent competition among mycoviruses in the same host.

To date five genera are approved by ICTV, and two additional genera were recently proposed. The ICTV sets species demarcation threshold at 50% identity for both CP and RdRp in the family *Totiviridae*. All five CrTV CPs shared amino acid identities below 50% to previously reported viruses in this family. Both phylogenetic analyses of the putative RdRp and CP revealed that CrTV1 was only distantly related to the members of the above seven genera (five approved and two proposed). CrTV1 RdRp was clustered with PpRV2, DcaRV17, UmV-H1, and RPaTV9 in the RdRp phylogenetic tree, but only CrTV1 and RpRV2 were clustered together in the CP phylogenetic tree. This is probably due to the latter two sharing fungi as their hosts [37]. CPs of DcaRV17, UmV-H1, and RPaTV9 were clustered together in one clade, much closer to the clade with *Giardiavirus* and *Insevirus* but distantly separated from the clade with CrTV1 and PpRV2. UmV-H1 and RPaTV9 likely established an additional genus in the family [8]. DcaRV17 was detected in diatom hosts [38]. Long-term adaptation to different host groups (fungi vs. diatom) might have caused their CPs to be phylogenetically more distant than their RdRps. CP is a structural protein with a function more related to host selection and host adaptation, while RdRp is a functional protein with replication domains more conserved across all different viruses. This may help explain differences in the phylogenetic relationships between CP- and RdRp-based analyses. In contrast, evolutionary relationships of four CrTVs with their closest homologs (CrTV2 vs. PsV2, CrTV3 vs. RPaTV5, CrTV4 vs. PsV3, and CrTV5 vs. RPaTV1a/PsV5) were very consistent between CP- and RdRp-based phylogenetic analyses. The hosts of CrTV, PsV, and RPaTV (*C. ribicola*, *Puccinia striiformis, Erysiphe trifoliorum*) are obligate biotrophs [8, 9]. The genus *Totivirus* infects a wide range of hosts, and totiviruses have been detected in other obligate biotrophs belonging to both the Basidiomycota and the Ascomycota, as well as higher plants, such as Ribes cultivars [10]. Although our study detected CrTVs in *C. ribicola* urediniospores collected from Ribes, low identities of RdRp sequences between CrTVs and the totivirus-like sequence from Ribes black currant indicted they were different members of Totivirus. Infection of closely related hosts may lead to viral CP and RdRp having similar amino acid changes and evolutionary rates. Based on available data regarding genome organization, amino acid identity, phylogeny, and host range and host speciality, we propose CrTV1 as a representative of a new genus *Cronartivirus* in the family *Totiviridae*. As more and more virus-like sequences have been identified in recent years, classification of the family *Totiviridae* may need to be revised [39–42]. Discovery of new dsRNA viruses with ancient origins may provide insight for understanding the evolution and distribution of RNA viruses [43].

Virus infection in fungi and parasites may be symptom-less or have phenotypic effects by association with either hypovirulence or hypervirulence. To look for potential relationships between CrTV infection and *C. ribicola* virulence, we surveyed the prevalence of five CrTVs across 33 rust samples. We found all of the fungal samples from the infected *P. albicaulis* trees were devoid of CrTV4 but abundantly infected by the other four CrTVs. Absence of CrTV4 in *C. ribicola*-infected *P. albicaulis*, as well as low frequency of CrTV4 in *C. ribicola*-infected *P. flexilis*, suggests that there may be a significant disadvantage for CrTV4-infected *C. ribicola* to infect and propagate in the two five-needle pine species well adapted at high elevations. The pine hosts may affect the success of a mycovirus in a fungus even after infection and colonization.

Furthermore, a significant association was detected between presence of CrTV4 and *C. ribicola vcr2* virulence, suggesting that there may be some epigenetic factors that caused changes in the pathogenicity of *C. ribicola*. Several case studies demonstrated that totiviruses play important roles in altering the fitness and virulence of the fungi and parasites. Expression of viral genome-encoded toxin in yeast and fungal species killed uninfected peers, providing the viral hosts with a survival advantage [44, 45]. LRV is associated with some benefits in survival advantages for its host parasite, such as reduced sensitivity to oxidative stress, and avoidance of immune destruction, increasing host-host parasite transmission [30]. On the other hand, the virulence level was decreased in *Beauveria bassiana* and *Bostrytis cinera* following infection by viruses, suggesting potential application of mycoviruses as bio-control agents against agricultural pests [46, 47]. The beneficial effects of totiviral infection on virulence or survival of their fungal hosts have been reported [48–50], and our data suggest that mycoviruses in *C. ribicola* may enhance virulence on host trees. We recognize that the sampling size in the present study was limited, and a fungal inoculation test to *P. albicaulis* or *P. flexilis* as well as a large survey of field infected trees are required to monitor CrTV4 accumulation in a future study. A future comparison of virus-free and virus-infected isogenic lines would conclusively prove any link between the presence of a specific virus and fungal virulence.

It is challenging to assess the effects of the virus infections on *C. ribicola* in the WPBR pathosystem because *invitro* culture of biotrophic rust fungi is very difficult, making it unfeasible to produce virus-free and virus-infected isogenic *C. ribicola* lines. To date, virulence in *C. ribicola* is determined by observation of canker development on pine trees with major gene resistance post rust infection [12]. Although genetic diversity of the fungal pathogen was likely limited when *C. ribicola* was introduced, 100 years of interaction with new hosts (especially those trees with major gene resistance) and environments has apparently begun to produce distinct fungal populations [51]. Given the economic and ecological significance of *C. ribicola* infections in five-needle pines, it would be worthwhile to investigate the presence of distinct mycoviruses in a large population of field rust-infected tree samples, exploring viral association with severity of canker development and tree survival. In addition to screening of genetic resistance in different five-needle pine populations [52, 53] and characterization of *C. ribicola* virulent factors [54], confirmation of hypo- or hyper-virulent effects of the viruses on *C. ribicola* may provide alternative tools for WPBR management.

## Conclusions

In the present work five novel dsRNA sequences were characterized in *C. ribicola*. Four (CrTV2-CrTV5) were identified as new members of the genus *Totivirus*, and the other (CrTV1) was proposed to be a representative of previously unclassified genus of the family *Totiviridae*. Both genome prevalence and transcript-related RNA expression analyses revealed dynamic changes of totiviruses over stages of the rust fungal life cycle across 33 *C. ribicola* samples. Significant association of CrTV4 with *C. ribicola* virulence suggests that mycoviruses are potential diagnostic markers for assessment of WPBR pathogenic diversity.

## Supplementary information

**Additional file 1: Figure S1.** Phylogenetic trees of the family Totiviridae based on the deduced amino acid sequences of capsid proteins. Sequences were aligned using Clustal Omega, and trees were generated using the maximum likelihood method with a bootstrap test of 100 iterations in MEGA 6 software. (a) Totiviruses grouping in the genus Totivirus. (b) Toti-like sequences potentially belonging to other genera of the family Totiviridae. Sequence identifications are the same as shown in Fig. 2.

**Additional file 2: Figure S2.** Pearson correlation analysis of fungal virulence and viral RNA levels of CrTV4 as detected by qRT-PCR. *Cronartium ribicola vcr2* samples were set at a virulence level of 1 (*N* = 6) and *avcr2* samples were set at a virulence level of 0 (*N* = 27).

**Additional file 3: Figure S3.** CrTV4 RNA levels measured by qRT-PCR in 33 *Cronartium ribicola* samples. Bars show average RNA levels of each virus with standard error of the mean (SEM). One-way Analysis of Variance (ANOVA) showed significant difference among seven types of samples (*P* = 0.014).

**Additional file 4: Figure S4.** Pearson correlation analysis of viral RNA levels as measured by RNA-seq analysis and qRT-PCR.

**Additional file 5: Table S1.** *Cronartium ribicola* samples collected in the present study. **Table S2.** Contigs of the *Cronartium ribicola* transcriptome with significant homologies to *Totiviruses* revealed by Blastx search. **Table S3.** Oligonucleotide sequences designed for PCR. **Table S4.** Open reading frames (ORFs) identified in the *C. ribicola* viral full-length genomes. **Table S5.** CDD search of putative *C. ribicola* Totivirus dsRNA genomes. **Table S6.** Pfam search of putative *C. ribicola* Totivirus dsRNA genomes. **Table S7.** Amino acid sequence identities among putative CrTV proteins and other members of the family Totiviridae.

## Data Availability

Sequence data were deposited in GenBank and materials were available with reasonable request.

## Abbreviations

CP: Capsid protein
CrTV: *Cronartium ribicola* totivirus
dsRNA: Double stranded RNA
ORF: Open reading frame
qRT-PCR: Quantitative reverse transcription-polymerase chain reaction
RACE: Rapid amplification of cDNA ends
RdRp: RNA-dependent RNA polymerase
WPBR: White pine blister rust

## References

[1] Nuss DL (2005). Hypovirulence: mycoviruses at the fungal–plant interface. Nat Rev Microbiol..

[2] Xie J, Jiang D (2014). New insights into mycoviruses and exploration for the biological control of crop fungal diseases. Annu Rev Phytopathol.

[3] Ghabrial SA, Castón JR, Jiang D, Nibert ML, Suzuki N (2015). 50-plus years of fungal viruses. Virol..

[4] King AM, Adams MJ, Lefkowitz EJ, editors. Virus Taxonomy: Ninth Report of the International Committee on Taxonomy of Viruses, vol. 9. San Diego: Elsevier; 2012.

[5] ICTV—International Committee on Taxonomy of Viruses (2014). Virus Taxonomy.

[6] Zhai Y, Attoui H, Mohd Jaafar F, Wang HQ, Cao YX, Fan SP, Sun YX, Liu LD, Mertens PP, Meng WS, Wang D, Liang G (2010). Isolation and full-length sequence analysis of Armigeres subalbatus totivirus, the first totivirus isolate from mosquitoes representing a proposed novel genus (*Artivirus*) of the family *Totiviridae*. J Gen Virol.

[7] Zhang P, Liu W, Cao M, Massart S, Wang X (2018). Two novel totiviruses in the white-backed planthopper, *Sogatella furcifera*. J Gen Virol.

[8] Kondo H, Hisano S, Chiba S, Maruyama K, Andika IB, Toyoda K, Fujimori F, Suzuki N (2016). Sequence and phylogenetic analyses of novel totivirus-like double-stranded RNAs from field-collected powdery mildew fungi. Virus Res.

[9] Zheng L, Lu X, Liang X, Jiang S, Zhao J, Zhan G, Liu P, Wu J, Kang Z (2017). Molecular characterization of novel totivirus-like double-stranded RNAs from *Puccinia striiformis* f. sp. tritici, the causal agent of wheat stripe rust. Front Microbiol.

[10] Cox S, Mayo MA, Jones AT (2000). The occurrence of dsRNA species in apparently healthy and virus-infected ribes cultivars, and evidence that one such species originates from a member of the virus family *Totiviridae*. Eur J Plant Pathol.

[11] Fins L, Byler JW, Ferguson D, Harvey AE, Mahalovich MF, McDonald GI, Miller D (2002). Return of the giants: restoring western white pine to the inland northwest. J Forest.

[12] Kinloch BB, Sniezko RA, Dupper GE (2004). Virulence gene distribution and dynamics of the white pine blister rust pathogen in western North America. Phytopathol.

[13] Liu J-J, Sturrock RN, Sniezko RA, Williams H, Benton R, Zamany A (2015). Transcriptome analysis of the white pine blister rust pathogen *Cronartium ribicola*: de novo assembly, expression profiling, and identification of candidate effectors. BMC Genomics.

[14] Liu J-J, Chan D, Xiang Y, Williams H, Li XR, Sniezko RA, Sturrock RN (2016). Characterization of five novel mitoviruses in the white pine blister rust fungus *Cronartium ribicola*. PLoS ONE.

[15] Marchler-Bauer A, Bo Y, Han L, He J, Lanczycki CJ, Lu S, Chitsaz F, Derbyshire MK, Geer RC, Gonzales NR, Gwadz M, Hurwitz DI, Lu F, Marchler GH, Song JS, Thanki N, Wang Z, Yamashita RA, Zhang D, Zheng C, Geer LY, Bryant SH (2017). CDD/SPARCLE: functional classification of proteins via subfamily domain architectures. Nucleic Acids Res.

[16] El-Gebali Sara, Mistry Jaina, Bateman Alex, Eddy Sean R, Luciani Aurélien, Potter Simon C, Qureshi Matloob, Richardson Lorna J, Salazar Gustavo A, Smart Alfredo, Sonnhammer Erik L L, Hirsh Layla, Paladin Lisanna, Piovesan Damiano, Tosatto Silvio C E, Finn Robert D (2018). The Pfam protein families database in 2019. Nucleic Acids Research.

[17] Tamura K, Stecher G, Peterson D, Filipski A, Kumar S (2013). MEGA6: molecular evolutionary genetics analysis version 6.0. Mol Biol Evol.

[18] Huang C-H, Lu CL, Chiu HT (2005). HPknotter: a heuristic approach for detecting RNA H-type Pseudoknots - offers a variety of tools including pknotsRG, PNOTS and NUPACK. Bioinformatics.

[19] Livak KJ, Schmittgen TD (2001). Analysis of relative gene expression data using real-time quantitative PCR and the 2(−Delta Delta C(T)) method. Methods.

[20] Liu J-J, Sniezko RA, Sturrock RN, Chen H (2014). Western white pine SNP discovery and high-throughput genotyping for breeding and conservation applications. BMC Plant Biol.

[21] Baeza M, Bravo N, Sanhueza M, Flores O, Villarreal P, Cifuentes V (2012). Molecular characterization of totiviruses in *Xanthophyllomyces dendrorhous*. Virol J.

[22] Wickner RB, Fujimura T, Esteban R (2013). Viruses and prions of *Saccharomyces cerevisiae*. Adv Virus Res.

[23] Plant EP, Jacobs KL, Harger JW, Meskauskas A, Jacobs AL, Baxter JL, Petrov AN, Dinman JD (2003). The 9-a solution: how mRNA pseudoknots promote efficient programmed −1 ribosomal frameshifting. RNA.

[24] Kim SN, Choi JH, Park MW, Jeong SJ, Han KS, Kim HJ (2005). Identification of the +1 ribosomal frameshifting site of LRV1-4 by mutational analysis. Arch Pharm Res.

[25] Wu B, Zhang X, Gong P, Li M, Ding H, Xin C, Zhao N, Li J (2016). *Eimeria tenella*: a novel dsRNA virus in *E. tenella* and its complete genome sequence analysis. Virus Genes.

[26] Maga JA, Widmer G, Lebowitz JH (1995). Leishmania RNA virus1-mediated cap-independent translation. Mol Cell Biol.

[27] Lee SE, Suh JM, Scheffter S, Patterson JL, Chung IK (1996). Identification of a ribosomal frame shift in Leishmania RNA virus1–4. J Biochem.

[28] Ro YT, Scheffter SM, Patterson JL (1997). Specific in vitro cleavage of a Leishmania virus capsid-RNA-dependent RNA polymerase polyprotein by a host cysteine-like protease. J Virol.

[29] Firth AE, Brierley I (2012). Non-canonical translation in RNA viruses. J Gen Virol.

[30] Hartley M-A, Ronet C, Zangger H, Beverley SM, Fasel N (2012). Leishmania RNA virus: when the host pays the toll. Front Cell Infect Microbiol.

[31] Huang S, Ghabrial SA (1996). Organization and expression of the double-stranded RNA genome of Helminthosporium victoriae 190S virus, a totivirus infecting a plant pathogenic filamentous fungus. Proc Natl Acad Sci U S A.

[32] Poulos BT, Tang KF, Pantoja CR, Bonami JR, Lightner DV (2006). Purification and characterization of infectious myonecrosis virus of penaeid shrimp. J Gen Virol.

[33] Pecman A, Kutnjak D, Gutiérrez-Aguirre I, Adams I, Fox A, Boonham N, Ravnikar M (2017). Next generation sequencing for detection and discovery of Plant viruses and Viroids: comparison of two approaches. Front Microbiol.

[34] Adams I, Fox A, Wang A, Zhou X (2016). Diagnosis of plant viruses using next-generation sequencing and metagenomic analysis. Current Research Topics in Plant Virology.

[35] Candresse T, Filloux D, Muhire B, Julian C, Galzi S, Fort G (2014). Appearances can be deceptive: revealing a hidden viral infection with deep sequencing in a plant quarantine context. PLoS One.

[36] Wang Y, Atta S, Wang X, Yang F, Zhou C, Cao M (2018). Transcriptome sequencing reveals novel Citrus bark cracking viroid (CBCVd) variants from citrus and their molecular characterization. PLoS One.

[37] Sasai S, Tamura K, Tojo M, Herrero M-L, Hoshino T, Ohki ST, Mochizuki T (2018). A novel non-segmented double stranded virus from an Arctic isolate of *Pythium polare*. Virol.

[38] Urayama S-I, Takaki Y, Nunoura T (2016). FLDS: a comprehensive dsRNA sequencing method for intracellular RNA virus surveillance. Microbes Environ.

[39] Shi M, Lin X-D, Tian J-H, Chen L-J, Chen X, Li C-X, Qin X-C, Li J, Cao J-P, Eden J-S, Buchmann J, Wang W, Xu J, Holmes EC, Zhang Y-Z (2016). Redefining the invertebrate RNA virosphere. Nature.

[40] Colmant AMG, Etebari K, Webb CE, Ritchie SA, Jansen CC, van den Hurk AF, Bielefeldt-Ohmann H, Hobson-Peters J, Asgari S, Hall RA (2017). Discovery of new orbiviruses and totivirus from *Anopheles* mosquitoes in eastern Australia. Arch Virol.

[41] Akinyemi IA, Wang F, Chang ZX, Wu Q (2018). Genome characterization of the newly identified maize-associated totivirus Anhui. Arch Virol.

[42] Huang Y, Guo X, Zhang S, Zhao Q, Sun Q, Zhou H, Zhang J, Tong Y (2018). Discovery of two novel totiviruses from *Culex tritaeniorhynchus* classifiable in a distinct clade with arthropod-infecting viruses within the family *Totiviridae*. Arch Virol.

[43] de Lima JGS, Teixeira DG, Freitas TT, Lima JPMS, Lanza DCF (2019). Evolutionary origin of 2A-like sequences in *Totiviridae* genomes. Virus Res.

[44] Wickner RB (1996). Double-stranded RNA viruses of *Saccharomyces cerevisiae*. Microbiol Rev.

[45] Schmitt MJ, Breinig F (2006). Yeast viral killer toxins: lethality and self-protection. Nat Rev Microbiol.

[46] Castro M, Kramer K, Valdivia L, Ortiz S, Castillo A (2003). A double-stranded RNA mycovirus confers hypovirulence-associated traits to *Botrytis cinerea*. FEMS Microbiol Lett.

[47] Dalzoto PR, Glienke-Blanco C, Kava-Cordeiro V, Ribeiro JZ, Kitajima EW, Azevedo JL (2006). Horizontal transfer and hypovirulence associated with double-stranded RNA in *Beauveria bassiana*. Mycol Res.

[48] Koltin Y, Day PR (1976). Inheritance of killer phenotypes and double-stranded RNA in *Ustilago maydis*. Proc Natl Acad Sci U S A.

[49] Bostian KA, Elliott Q, Bussey H, Burn V, Smith A, Tipper DJ (1984). Sequence of the preprotoxin dsRNA gene of type I killer yeast: multiple processing events produce a two-component toxin. Cell.

[50] Ives A, Ronet C, Prevel F, Ruzzante G, Fuertes-Marraco S, Schutz F, Zangger H, Revaz-Breton M, Lye L-F, Hickerson SM, Beverley SM, Acha-Orbea H, Launois P, Fasel N, Masina S (2011). Leishmania RNA virus controls the severity of *Mucocutaneous Leishmaniasis*. Science.

[51] Richardson BA, Klopfenstein NB, Zambino PJ, McDonald GI, Geils BW, Carris LM (2008). Influence of host resistance on the genetic structure of the white pine blister rust fungus in the western United States. Phytopathol.

[52] Geils BW, Hummer KE, Hunt RS (2010). White pines, Ribes, and blister rust: a review and synthesis. Forest Pathol.

[53] Sniezko R, Smith J, Liu J-J, Hamelin R (2014). Genetic resistance to fusiform rust in southern pines and white pine blister rust in white pines—a contrasting tale of two rust pathosystems—current status and future prospects. Forests.

[54] Ma Z, Liu JJ, Zamany A (2019). Identification and functional characterization of an effector secreted by *Cronartium ribicola*. Phytopathol.

