# Supplementary figures and images for: Characterization of Cronartium ribicola dsRNAs reveals novel members of the family Totiviridae and viral association with fungal virulence
Source: Virol J. 2019 Oct 17;16:118. doi: 10.1186/s12985-019-1226-5 (PMC6796417; doi:10.1186/s12985-019-1226-5)

## Slide 1
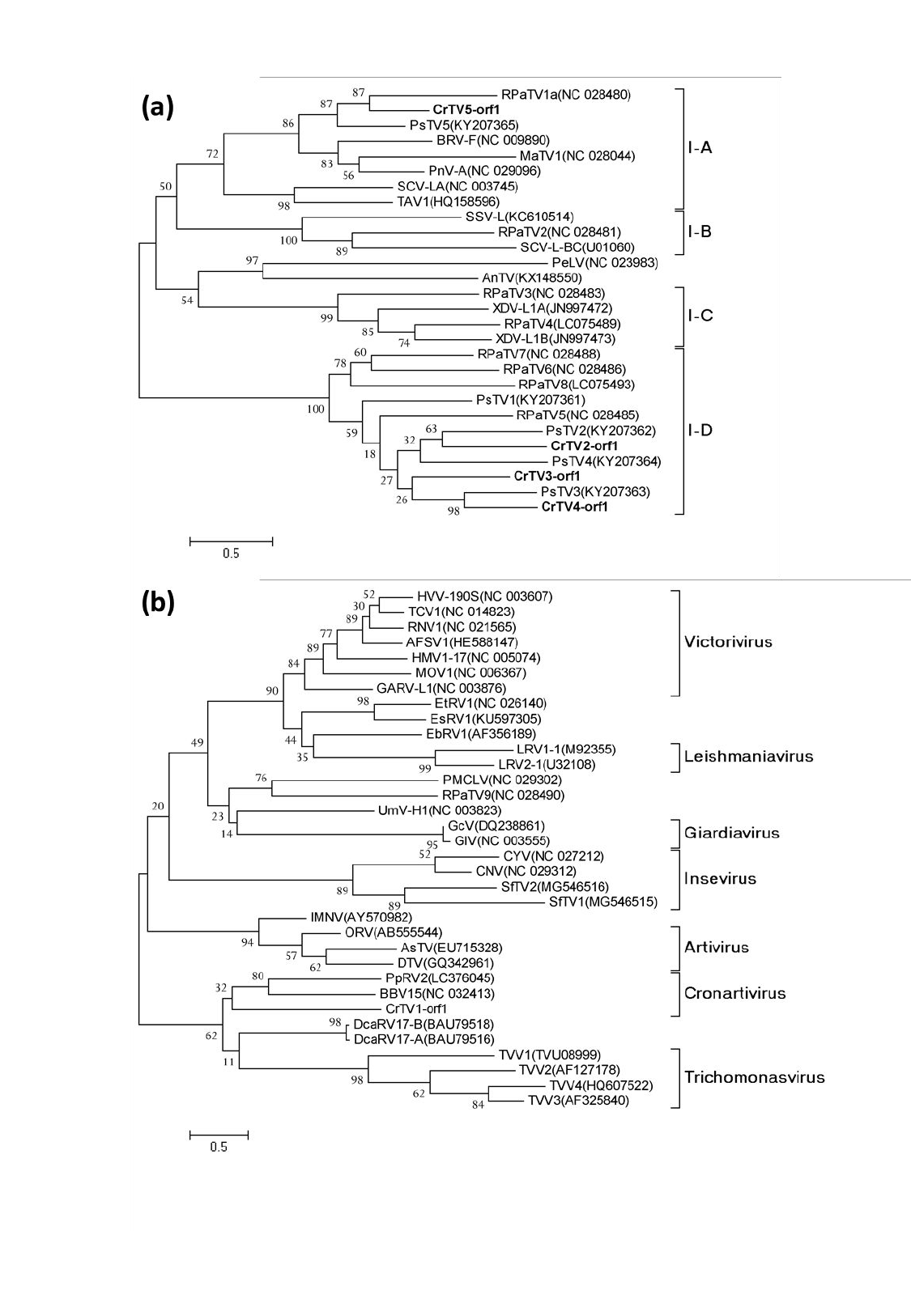

Supplement: Supplementary file 1 — Additional file 1: Figure S1. Phylogenetic trees of the family Totiviridae based on the deduced amino acid sequences of capsid proteins. Sequences were aligned using Clustal Omega, and trees were generated using the maximum likelihood method with a bootstrap test of 100 iterations in MEGA 6 software. (a) Totiviruses grouping in the genus Totivirus. (b) Toti-like sequences potentially belonging to other genera of the family Totiviridae. Sequence identifications are the same as shown in Fig. 2. [file 12985_2019_1226_MOESM1_ESM.pptx]

## Slide 1
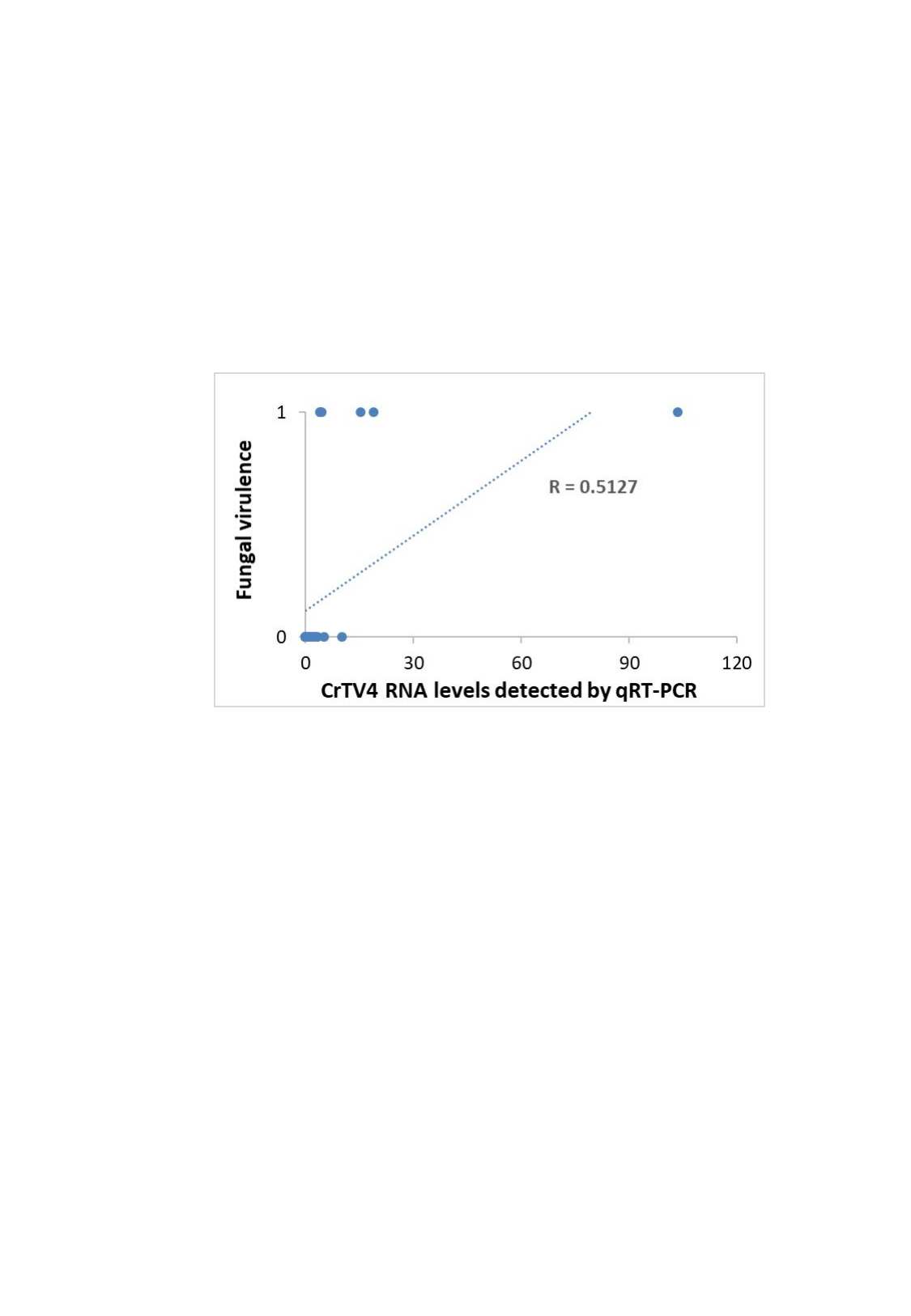

Supplement: Supplementary file 2 — Additional file 2: Figure S2. Pearson correlation analysis of fungal virulence and viral RNA levels of CrTV4 as detected by qRT-PCR. Cronartium ribicola vcr2 samples were set at a virulence level of 1 (N = 6) and avcr2 samples were set at a virulence level of 0 (N = 27). [file 12985_2019_1226_MOESM2_ESM.pptx]

## Slide 1
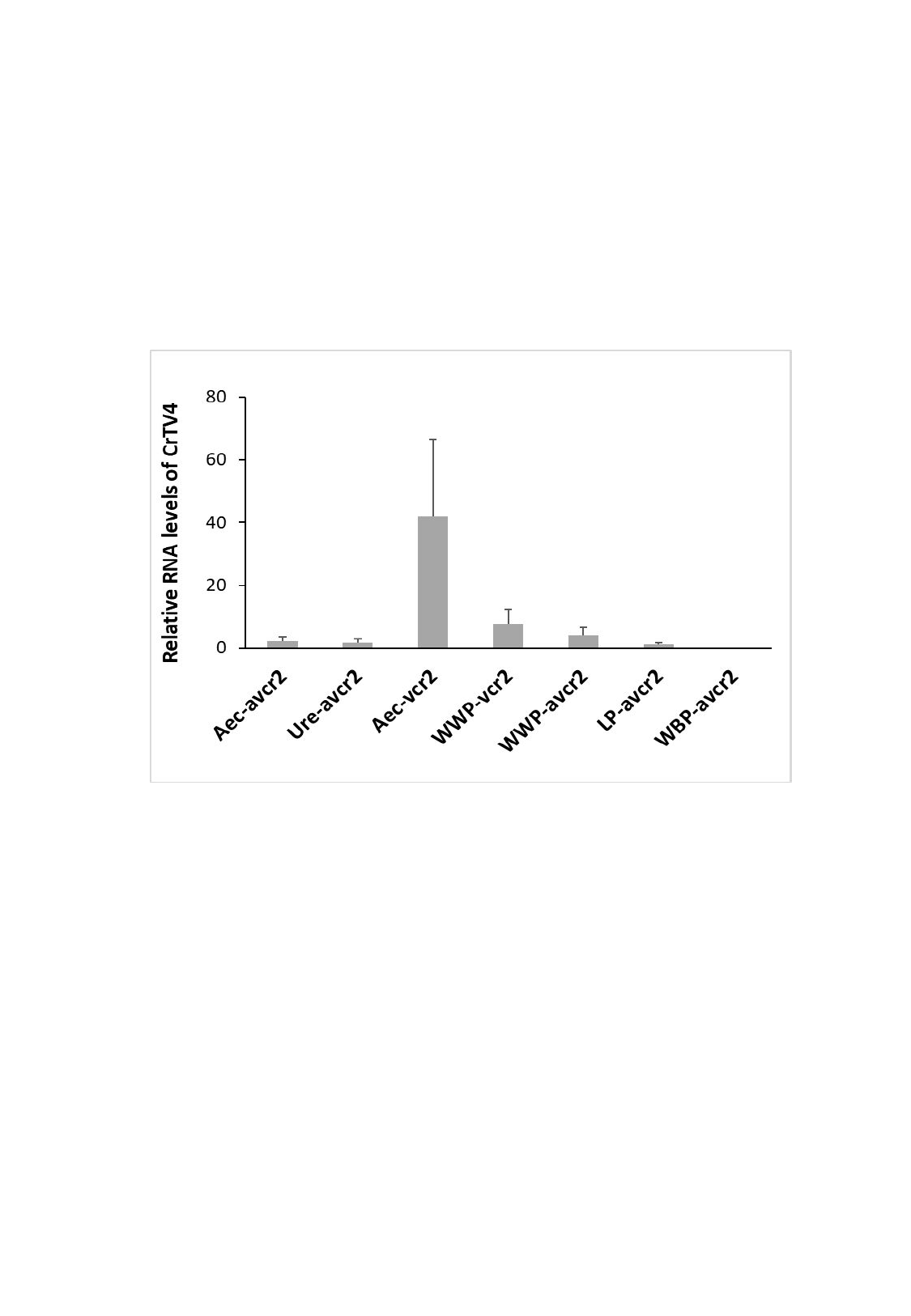

Supplement: Supplementary file 3 — Additional file 3: Figure S3. CrTV4 RNA levels measured by qRT-PCR in 33 Cronartium ribicola samples. Bars show average RNA levels of each virus with standard error of the mean (SEM). One-way Analysis of Variance (ANOVA) showed significant difference among seven types of samples (P = 0.014). [file 12985_2019_1226_MOESM3_ESM.pptx]

## Slide 1
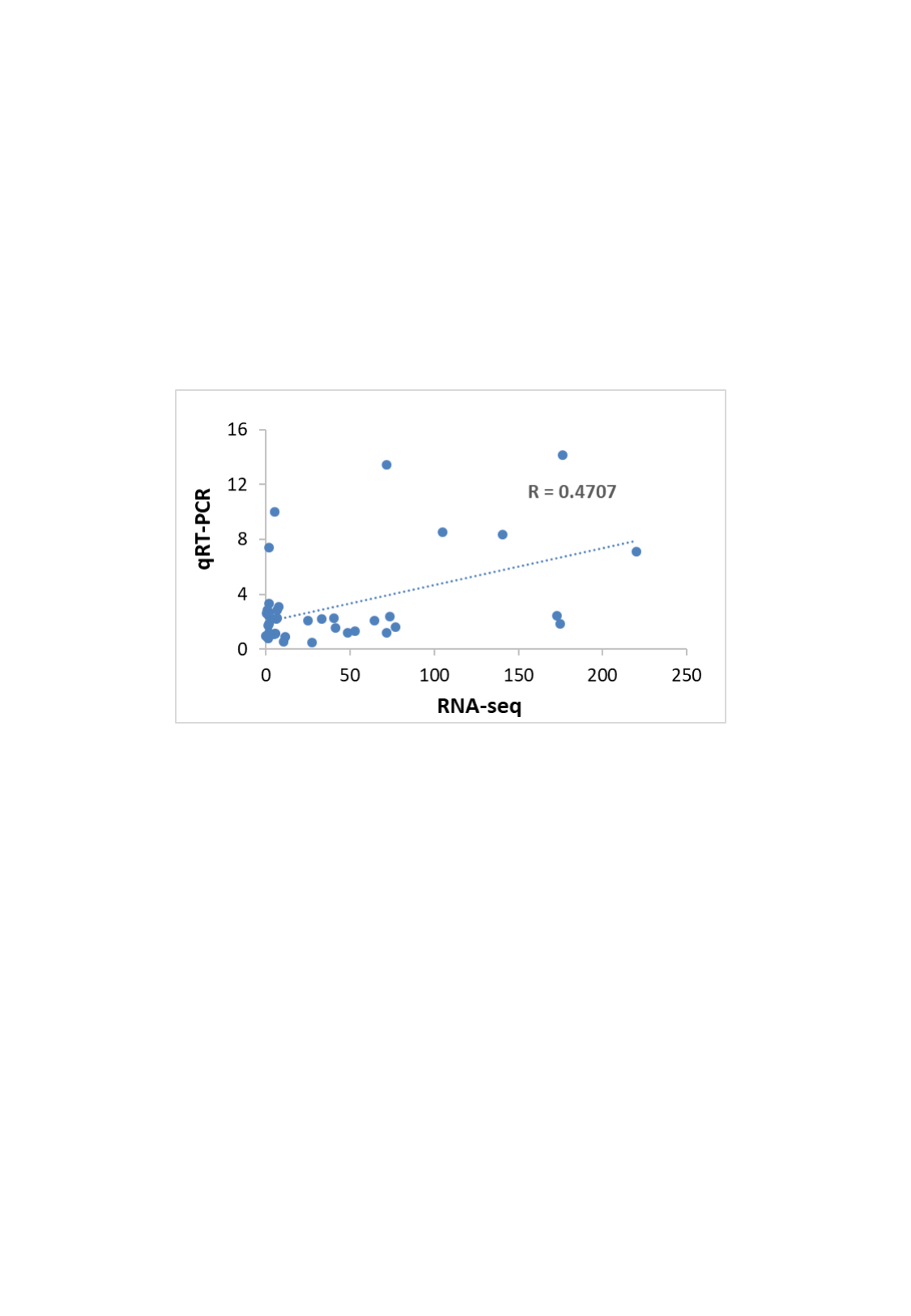

Supplement: Supplementary file 4 — Additional file 4: Figure S4. Pearson correlation analysis of viral RNA levels as measured by RNA-seq analysis and qRT-PCR. [file 12985_2019_1226_MOESM4_ESM.pptx]
